# Genetic adaptation to high altitude in the Ethiopian highlands

**DOI:** 10.1186/gb-2012-13-1-r1

**Published:** 2012-01-20

**Authors:** Laura B Scheinfeldt, Sameer Soi, Simon Thompson, Alessia Ranciaro, Dawit Woldemeskel, William Beggs, Charla Lambert, Joseph P Jarvis, Dawit Abate, Gurja Belay, Sarah A Tishkoff

**Affiliations:** 1 Department of Genetics, University of Pennsylvania, 415 Curie Boulevard, Philadelphia, PA 19104, USA; 2 Department of Biology, Addis Ababa University, PO Box 1176, Addis Ababa, Ethiopia; 3 Cold Spring Harbor Laboratory, 1 Bungtown Road, Cold Spring Harbor, NY 11724, USA; 4 Department of Biology, University of Pennsylvania, 433 S. University Avenue, Philadelphia, PA 19104, USA

## Abstract

**Background:**

Genomic analysis of high-altitude populations residing in the Andes and Tibet has revealed several candidate loci for involvement in high-altitude adaptation, a subset of which have also been shown to be associated with hemoglobin levels, including *EPAS1, EGLN1*, and *PPARA*, which play a role in the HIF-1 pathway. Here, we have extended this work to high- and low-altitude populations living in Ethiopia, for which we have measured hemoglobin levels. We genotyped the Illumina 1M SNP array and employed several genome-wide scans for selection and targeted association with hemoglobin levels to identify genes that play a role in adaptation to high altitude.

**Results:**

We have identified a set of candidate genes for positive selection in our high-altitude population sample, demonstrated significantly different hemoglobin levels between high- and low-altitude Ethiopians and have identified a subset of candidate genes for selection, several of which also show suggestive associations with hemoglobin levels.

**Conclusions:**

We highlight several candidate genes for involvement in high-altitude adaptation in Ethiopia, including *CBARA1, VAV3, ARNT2 *and *THRB*. Although most of these genes have not been identified in previous studies of high-altitude Tibetan or Andean population samples, two of these genes (*THRB *and *ARNT2*) play a role in the HIF-1 pathway, a pathway implicated in previous work reported in Tibetan and Andean studies. These combined results suggest that adaptation to high altitude arose independently due to convergent evolution in high-altitude Amhara populations in Ethiopia.

## Background

Modern humans migrated out of Africa at least 60,000 years ago and subsequently colonized a diverse array of environments, including regions located at high altitude (> 2,500 meters). The three most dramatic examples of long-term high-altitude residence are populations living on the Tibetan Plateau, the Andean Altiplano, and the Ethiopian Highlands. Much of the reported work to date has focused on the characterization of biological adaptation to high altitude, predominately in Asian and South American populations, resulting in an extensive body of work (reviewed in [1-3]). A portion of this research has identified particular physiological traits in high-altitude Asian and South American populations that appear to mitigate the impact of hypoxia at high altitude [1,2].

Due to reduced oxygen levels at high altitude, two physiological phenotypes involved in oxygen transport that are commonly studied in high-altitude populations are hemoglobin levels and oxygen saturation in the blood. Concentrations of hemoglobin are elevated in high-altitude Andean populations relative to high-altitude Asian and African populations as well as low-altitude populations, and oxygen saturation is reduced in high-altitude Andeans as well as in Tibetans (who do not have increased hemoglobin levels) [4]. Oxygen saturation has been shown to have moderate heritability (*h^2 ^*= 0.65) in Tibetan populations, and hemoglobin levels have been shown to have high heritability (*h^2 ^*= 0.89) in both Tibetan and Andean populations [5]. In addition, work by Beall *et al*. [6] has demonstrated strong selective pressure favoring high-altitude Tibetan women with high oxygen saturation of hemoglobin, who have more than twice as many surviving offspring as women with low oxygen saturation of hemoglobin. Furthermore, Julian *et al*. [7] studied pregnant Andean and European women at high and low altitude and have shown that Andean ancestry confers a protective effect during pregnancy involving improved uterine blood flow and fetal growth at high altitude. Thus, physiological traits that reduce hypoxic stress, and the underlying genetic factors influencing these traits, are likely to be common in long-term high-altitude residents.

No distinguishing physiological traits, however, have been identified in high-altitude Ethiopians [4,5]. Hemoglobin levels and oxygen saturation in high-altitude Ethiopians living in the Ambaras area (3,530 meters) have been reported to not significantly differ from those in low-altitude residents in the United States [4,5]. The variation in high-altitude physiology among populations originating from different geographic regions suggests that there may be different biological mechanisms playing a role in high-altitude adaptation in these populations.

In addition, there have been several recent genomic studies of high-altitude Andean and Tibetan population samples that have identified a set of candidate genes (including *EPAS1, EGLN1*, and *PPARA*) thought to contain variants that play a role in physiological adaptation to high altitude [8-13]. To date, however, there have been no genomic studies reported on the populations residing in the Ethiopian Highlands. Here we present the results of a genome-wide analysis of over 1 million SNPs genotyped with the Illumina 1M duo genotyping chip in a sample of high-altitude Amhara individuals (living at 3,202 meters above sea level; *n *= 28) and low altitude Aari and Hamer individuals (living at < 1,500 meters above sea level; *n *= 19) residing in Ethiopia. We show that there are significantly higher hemoglobin levels in high- (3,200 meters) relative to low-altitude (< 1,500 meters) Ethiopian residents. We performed genome-wide analyses to identify regions of the genome that are the strongest candidates for recent positive selection in a high-altitude Amhara population sample, identified significantly enriched pathways, and tested the strongest candidates for selection for genotype/phenotype associations with hemoglobin levels. We identified several candidates for involvement in high-altitude adaptation, including *CBARA1, VAV3, ARNT2 *and *THRB*.

## Results

### Population structure

**Figure 1:**
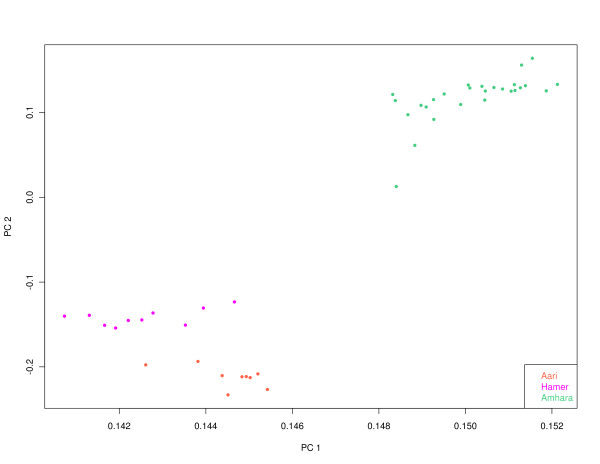
**Principal components analysis of Amhara, Aari and Hamer individuals**. Principal component (PC)1 (x-axis) versus PC2 (y axis). The Amhara are displayed in green, the Aari in orange, and the Hamer in magenta.

We first performed principal components analyses on a pruned set of 324,962 genome-wide SNPs (R^2 ^cutoff = 0.5), to characterize the pattern of individual clustering in the sample set. As shown in Figure 1, PC1 (which accounts for 27.7% of the total variance) and PC2 (which accounts for 3.05% of the total variance) both separate the Amhara and Aari/Hamer population samples from each other. Furthermore, the Aari and Hamer cluster closely to each other relative to the Amhara population samples. Therefore, in downstream analyses we combined the Aari and Hamer population samples into a single population sample that we hereafter refer to as Omotic since both the Aari and Hamer speak languages that belong to the Omotic branch of the Afroasiatic language family.

### Genome-wide tests of neutrality

We were interested in identifying the subset of SNPs that were highly differentiated between the Amhara and Omotic population samples, because these SNPs are likely to be enriched for variants (or to be in linkage disequilibrium (LD) with variants) that have been subjected to regionally restricted positive selection. Therefore, we calculated pairwise F_ST _[14] on the SNP data generated from the Omotic population sample and the unrelated Amhara population sample; Table S1 in Additional file 1 includes the SNPs in the top 0.1% of the empirical distribution (F_ST _> 0.33). The top F_ST _regions (including 100 kb up- and downstream of each candidate SNP) were not significantly enriched for any Panther pathways or for hypoxia inducible factor (HIF)-1 pathway genes after correcting for multiple testing.

**Figure 2:**
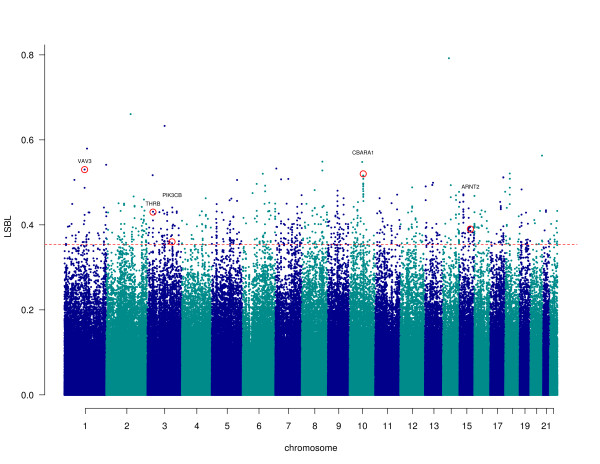
**Genome-wide distribution of LSBL values**. The chromosomes are plotted along the x-axis, and the LSBL values are plotted along the y-axis. Chromosomes are colored alternately in dark blue and light blue, and the 99.9% percentile is denoted with a dashed red line. The top candidate gene SNPs are circled in red and the gene names are labeled above.

In addition, we utilized the HapMap phase three data from the unrelated Yoruba population samples from Ibadan Nigeria (YRI) [15] (*n *= diploid individuals = 113) to calculate a locus-specific branch length (LSBL) [16] value for each of the polymorphic SNPs in the merged dataset (878,625 SNPs) (Figure 2). This three-population test identifies variants that have highly differentiated allele frequencies in each population sample relative to the other two. We can, therefore, identify the set of SNPs that are the strongest candidates for regionally restricted positive selection in the Amhara. Table S2 in Additional file 1 contains the SNPs in the top 0.1% of the empirical distribution (LSBL values > 0.36). The top results from our analysis include several candidate loci that have biological functions related to lung injury and/or response to hypoxia. These loci include *CBARA1, ARHGAP15*, and *RNF216*. CBARA1 regulates calcium uptake by the mitochondria [17], ARHGAP15 may be involved in survival after acute lung injury [18], and RNF216 encodes an enzyme that inhibits NF-kappa B activation pathways [19], which are involved in HIF-α induction [20]. In addition, we performed a pathway enrichment analysis using the Panther Classification System tools [21] on the top LSBL candidate genes (100 kb up- and downstream) to identify pathways that are overrepresented. We identified three pathways with significant *P*-values after correction for multiple testing: metabotropic glutamate receptor group III pathway (P_cor _= 2.8 × 10^-02^), beta1 adrenergic receptor signaling pathway (P_cor _= 3.7 × 10^-02^), and beta2 adrenergic receptor signaling pathway (P_cor _= 3.7 × 10^-02^). HIF-1 pathway genes were not significantly overrepresented in our top LSBL results.

**Figure 3:**
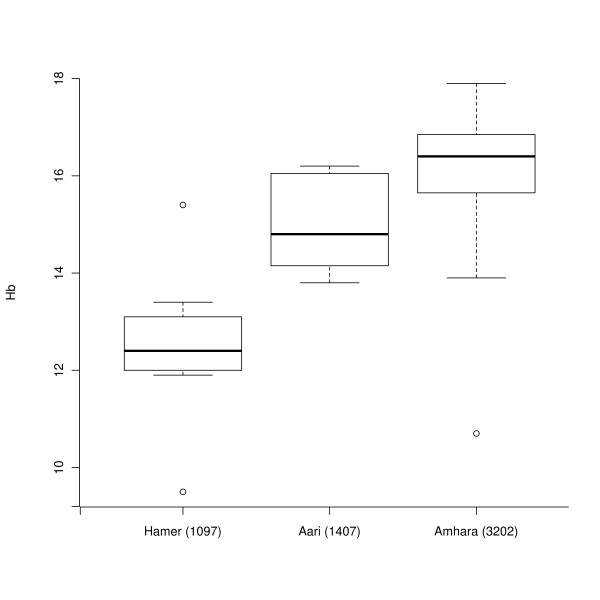
**Boxplot of hemoglobin levels estimated in the Amhara, Aari and Hamer population samples**. Each population sample along with the altitude in meters at which the sample was collected is shown on the x-axis, and the hemoglobin (Hb) levels are plotted on the y-axis.

We employed the integrated haplotype score (iHS) [22] to identify regions of the Amhara genomes that exhibit patterns of variation consistent with a recent selective sweep. The most extreme (top 0.1%) absolute iHS values (> 3.483736; Table S3 in Additional file 1) are in regions containing several genes that have plausible biological functions that could play a role in local adaptation, including genes involved in diet and metabolism and immune function. Indeed, differences in disease exposure related to altitude are likely to result in differences in selective pressures between the high- and low-altitude residents as previously noted (for example, [3]). These candidates include *SYNJ2*, which is involved in the phosphatidylinositol signaling pathway, and is upregulated after exposure to botulinum neurotoxins in SH-SY5Y cells [23], *NAT2*, which is involved in the metabolism of drugs used to treat tuberculosis [24], and *AIMP1*, which encodes a protein that is involved in the control of angiogenesis, inflammation, wound healing, and glucose homeostasis [25]. *AIMP1 *was also in the most extreme iHS results in the Omotic analysis, the top 0.1% of which are presented in Table S4 in Additional file 1. We performed a test of pathway enrichment using the Panther Classification System tools [21] on the top 0.1% of iHS results to identify pathways that are overrepresented. We identified two Panther pathways that were significantly overrepresented after correction for multiple testing: cadherin signaling (P_cor _= 2.8 × 10^-04^) and Wnt signaling (P_cor _= 2.1 × 10^-02^). Interestingly, cadherin 1 was previously implicated in a study of high-altitude adaptation in Andeans [8].

Additionally, we ran the cross-population composite likelihood ratio (XP-CLR) test on our dataset [26]. XP-CLR is a multi-locus sliding window test that identifies regions of the genome that are differentiated between populations. The regions with the XP-CLR values in the top 0.1% of the empirical distribution (XP-CLR > 5.874027) include *BCL11A*, which influences fetal hemoglobin levels [27], *CBARA1 *(which was also identified in the LSBL analysis) and *VAV3*, which induces GTPase activity [28] and is involved in angiogenesis [29] (Table S5 in Additional file 1). Interestingly, *VAV3 *is one of 14 genes that were identified in the top 0.1% of all four genome-wide scans for selection (Table S6 in Additional file 1). We found no significant enrichment of HIF-1 pathway genes or any Panther pathway in our top XP-CLR results after correcting for multiple testing.

### Phenotypic analyses of hemoglobin levels

In vertebrates, hemoglobin carries oxygen in red blood cells and is therefore a potential target for selection at high altitude. Moreover, previous work reported on Asian and American populations has shown strong heritability of hemoglobin levels (*h^2 ^*= 0.89) [5] and has documented significant variability in hemoglobin levels among high-altitude residents in South America relative to high-altitude Asian and African residents as well as low-altitude United States residents [5]. We were, therefore, interested in characterizing hemoglobin level variation among high-altitude and low-altitude Ethiopians. We measured hemoglobin levels in the field from 28 Amhara men (living at 3,202 meters), 8 Aari men (living at 1,407 meters) and 7 Hamer men (living at 1,097 meters). We observed a significant increase in hemoglobin levels in the Amhara (median = 16.4 grams per deciliter (g/dl)) relative to the Aari (median = 14.8 g/dl) and Hamer (median = 12.4 g/dl) population samples (*P *= 0.0003; Figure 3).

We restricted our association testing to the top 0.1% of the Amhara LSBL SNPs and the SNPs in the surrounding 100 kb region because these regions are likely to contain functional loci that play a role in adaptation to high altitude. We used EMMAX, a mixed model method that controls for population structure, to perform our association testing, and we used age and altitude as covariates [30]. Although none of the SNPs reach statistical significance after correction for multiple testing, our top results have -log10 *P*-values of 2 or higher (Table S7 in Additional file 1) and include two genes with biological relationships to HIF-1: *THRB *(*P *= 0.0017) and *ARNT2 *(*P *= 0.0018). ARNT2 is directly involved in the HIF-1 pathway, whereas *THRB *is required for HIF expression in hepatic cells [31,32]. As shown in Additional file 2, individuals with two copies of the *THRB *rs826216 C allele (derived) have the highest hemoglobin levels.

### Replication of selection and association signals in high-altitude Tibetan and Andean populations

Three genes have been previously implicated in high-altitude adaptation in Tibetan populations (*EPAS1, EGLN1*, and *PPARA*) [9,11,12]. *EGLN1 *has also been implicated as a candidate target of selection in Andean populations [13]. We were, therefore, interested in whether any of these genes were identified in our genome-wide scans of selection. While *EPAS1 *and *EGLN1 *were not implicated in any of our genome-wide scans of selection, *PPARA *was identified in our between-population XP-CLR (Table S5 in Additional file 1) test as well as in the within-population Amhara (Table S3 in Additional file 1) and Omotic (Table S4 in Additional file 1) iHS tests. We were additionally interested in whether variation at these loci is associated with hemoglobin levels in Ethiopians. Our results demonstrate that *PPARA *and *EPAS1 *both contain SNPs with marginal associations with hemoglobin (rs4253712, *P *= 0.025 and rs13412887, 0.027, respectively). These results are consistent with the possibility that variation at these loci may also play a role in adaptation to high altitude in the Ethiopian population.

## Discussion

Previous genomic studies involving high-altitude residents of Asia and South America have identified three candidate genes (*EPAS1, EGLN1*, and *PPARA*) that exhibit patterns of SNP variation consistent with recent positive selection [8-13]. In addition, variation at these loci has been shown to be associated with hemoglobin levels in high-altitude Asian population samples [9,11,12]. Furthermore, all three of these candidate genes are involved in the HIF-1 pathway cascade that is initiated in response to hypoxic environmental conditions and regulates oxygen homeostasis in humans and other mammals [31]. While *PPARA *was identified as a target of selection in our Ethiopian population sample, we have identified several additional candidate genes for involvement in high-altitude adaptation in the Amhara, two of which also play a role in the HIF-1 pathway.

Our combined genome-wide scans for selection identified several candidate genes that may have biological functions related to hypoxia. Of note is the gene containing two out of the most extreme ten XP-CLR SNPs, *CBARA1 *(also known as *MICU1*), which also contains six SNPs in the top 20 of the LSBL results. One possible explanation for why *CBARA1 *may not have been distinguished by the iHS test is that the region includes multiple extended haplotypes in the Amhara (shown in grey, red, green, and aqua in Additional file 3) as well as in the low altitude populations. However, we also observe a region of high haplotype homozygosity in the Amhara that encompasses a 500 kb region of high LSBL SNPs (shown in black in Additional file 3). If we consider a 3 SNP core centered in the signal of high LSBL in the Amhara, it is present at 48.2% frequency in the Amhara and 2.6% frequency in the lower altitude populations. This pattern of haplotype variation is consistent with positive selection on an Amhara-specific variant that is being tagged by the high frequency haplotype (shown in black in Additional file 3). Furthermore, *in vitro *work conducted in HeLa cells demonstrates that CBARA1 localizes to the mitochondria and is required for Ca^2+ ^uptake, which plays a role in ATP production and cell death [17]. Therefore, CBARA1 is a potential candidate for involvement in the way in which HIF-1 regulates mitochondrial metabolism, which is argued to play a critical role in the response to hypoxic conditions [33]. Another candidate gene for involvement in high-altitude adaptation is *VAV3*, one of the 14 genes identified in all four genome-wide scans of selection. VAV3 regulates GTPase activity *in vitro *[28] and is involved in angiogenesis *in vivo *[29]. Furthermore, hypoxia induces angiogenesis in adulthood, and this process is largely initiated by HIF-1 [34].

We have additionally identified two candidate genes, *ARNT2 *and *THRB*, that exhibit patterns of SNP variation consistent with positive selection, contain variation that is associated with hemoglobin levels, and are involved with the HIF-1 pathway cascade that is initiated under hypoxic conditions. THRB is expressed in hepatic cells where it has been shown to form a heterodimer with retinoid × receptor (RXR) that is required for HIF expression [31,32]. During fetal development, the liver is the primary source of erythropoietin, and HIF regulates the production of erythropoietin, which is required for red blood cell production [31]. HIF1a and ARNT2 (aka HIF1b) form a heterodimer that is present in most cells, and appears to play a general role in the response to hypoxia [31]. Moreover, EPAS1 (aka HIF2a; which has highly conserved functional domains with HIF1a) and ARNT2 form a heterodimer that is expressed in fetal lung [31]. In addition, *ARNT2 *was previously implicated in high-altitude adaptation in Andeans [8].

Although we have identified a number of candidate genes that may play a role in adaptation to high altitude in Ethiopians, we are limited by our modest sample size as well as by the ascertainment and coverage of our SNP dataset. The SNPs included in the Illumina 1M duo genotyping chip were primarily selected based on identification and patterns of LD in non-Africans. Thus, due to relatively lower levels of LD in African populations, we may not have the power to detect all of the genetic variants involved in high-altitude adaptation in the Amhara. Indeed, whole-genome sequencing may be necessary to identify population-specific variants in the high-altitude Amhara. Additional functional analysis is also necessary to more fully understand the roles that variation at these loci play in high-altitude adaptation.

## Conclusions

Our analysis has produced several candidates for involvement in high-altitude physiology, including *CBARA1, VAV3, ARNT2 *and *THRB*, three of which have not been previously implicated in genome-wide high-altitude studies. Each of these genes has a biological function that may play a role in the response to hypoxia, and two of them (*THRB *and *ARNT2*) play a role in the HIF-1 pathway, which was previously implicated in Tibetan and Andean studies [8-13]. Our combined results suggest that the genes and genetic variants contributing to high-altitude adaptation in Ethiopia are largely distinct from other high-altitude regions and arose independently through convergent evolution due to the strong selective force of hypoxia.

## Materials and methods

### Samples

We obtained institutional review board approval for this project from the University of Pennsylvania. Prior to sample collection, we obtained informed consent from all research participants, and permits from the Federal Democratic Republic of Ethiopia Ministry of Science and Technology National Health Research Ethics Review Committee. We focused on a sample of 28 male individuals living in the Amhara region in Debele, which is near Debre Birhan, Ethiopia, at 3,203 meters altitude for the current analysis (females were excluded because the menstrual cycle and pregnancy can influence hemoglobin levels). The Amhara population speaks a language that belongs to the Semitic branch of the Afroasiatic language family [35]. Our study also included a comparison low-altitude (< 1,500 meters) population sample of nine individuals living in Gieza (the Aari), Ethiopia and ten individuals living in Dimeka (the Hamer), Ethiopia. Both the Aari and Hamer speak languages that belong to the Omotic branch of the Afroasiatic language family [35]. Additional file 4 displays the locations where the field work was conducted. In the field, 6 ml of blood was collected and white cells were isolated from whole blood with a salting out procedure modified from [36]. DNA was extracted in the lab with a Gentra Purgene DNA extraction kit (Qiagen Inc., Valencia, CA, USA). Hemoglobin levels were measured in the field using a HemoCue Hb201+ analyzer with HemoCue hemoglobin cuvettes.

### Gentoyping

DNA samples were genotyped using the Illumina 1M duo SNP array and markers that had at least 95% complete data (1,074,966 SNPs) were used for further analysis. All of the 47 samples had high call rates (> 95%), and the software package PLINK [37] was used to estimate relatedness among individuals. We identified one Amhara individual with a pi-hat value of over 0.25, and excluded this sample from all of the analyses that require assumptions of unrelatedness, such as the genome-wide scans for selection.

### Data analysis

#### PCA

We performed a principal components analysis as described in McVean [38] using R [39] without scaling on a subset of 324,962 pruned SNPs (R^2 ^cutoff = 0.5; using Plink [37]) to evaluate the pattern of genetic structure among the Amhara (*n *= 28), Aari (*n *= 9) and Hamer (*n *= 10) population samples. Since the Aari and Hamer population samples were tightly clustered in the principal components analysis, we combined them into a single Omotic population sample for the subsequent analyses described below.

#### Genome-wide tests of neutrality involving population structure

We were interested in identifying variants that are unusual in the high-altitude Amhara relative to low altitude Africans to construct a list of candidates enriched for SNPs involved in high-altitude adaptation. We first calculated pairwise F_ST _[14] between the unrelated Amhara individuals (pi-hat < 0.25; *n *= 27) and the combined unrelated Omotic population individuals (pi-hat < 0.25; *n *= 19). The distribution of pairwise F_ST _results can be seen in Additional file 5. In addition, we merged our SNP data with the data from the unrelated HapMap Yoruba from Ibadan Nigeria (YRI) population samples [15] and calculated a LSBL value for the Amhara [16]. We chose two African populations that are more closely related to our high-altitude Ethiopian population than Europeans or Asians to minimize the number of regions at which they are likely to differ due to demography (including the severe bottleneck associated with the out of Africa migration) rather than recent positive selection. This test allows us to identify SNPs with allele frequencies that are unusual in the Amhara relative to both the Omotic and Yoruba population samples. The distribution of LSBL results can be seen in Additional file 6. We additionally repeated our LSBL analysis using the unrelated HapMap CEPH population samples [15] in place of the Yoruba samples and found similar results in the Amharic population (Table S8 in Additional File 1).

#### Linkage disequilibrium-based genome-wide tests of neutrality

We also utilized a genome-wide test of neutrality that incorporates patterns of LD, since recent selective events often perturb neutral patterns of LD. We used the software package fastPHASE version 1.4 to infer phase [40], and we performed the iHS test [22]. We generated a fine-scale recombination map relevant to the African populations with LDhat version 2.1 [41]. Individuals used to generate the recombination map were 100 unrelated samples, 25 males and 25 females, each from two populations in HapMap3 release 2: the Yoruba from Ibadan, Nigeria (YRI) and the Luhya from Webuye, Kenya (LWK) [15]. We used genome-wide sequence data from several non-human primates (chimpanzee, orangutan, and rhesus macaque) downloaded from the UCSC Genome Browser website [42] to establish the ancestral allele for each of the SNPs included in our iHS analysis. Approximately 5% of the SNPs in our data could not be assigned an unambiguous ancestral state, and were removed prior to our iHS analysis. In addition, SNPs with minor allele frequencies less than 5% in either the Amhara or Aari/Hamer population samples were removed from the phased dataset used in the iHS analysis in agreement with recent publications (for example, [22]). The un-standardized scores returned by the iHS binary executable were adjusted such that all scores had zero means and unit variances with respect to SNPs with similar derived allele frequencies (for iHS, as described in [22]). The distribution of iHS results can be seen in Additional file 7. We considered all of the results (for iHS we took the absolute values) in the top 0.1% of the distribution to be the top candidates.

We additionally performed the XP-CLR test, which is more robust to selection from standing variation and to SNP ascertainment bias [26]. Using the recombination map described above, we estimated a genetic map in Morgan units using an effective population size (Ne) of 15,700, consistent with the estimation in [43]. We then ran the XP-CLR software package [26] with 0.005 cM sliding windows and a between window distance of 5 kb. The distribution of XP-CLR results can be seen in Additional file 8.

We tested for significantly over-represented Panther biological pathways [21]. Previous work has shown the HIF-1 pathway to be especially relevant to the study of high-altitude physiology. Therefore, in addition to the inclusion of the HIF-1 pathway (P00030) in the Panther analysis, and similar to previous studies [9], we investigated whether or not our top candidates for positive selection include an expanded set of HIF-1 pathway genes. We chose the most inclusive set of HIF-1 pathway genes as described in [9]. For each genome-wide scan of selection we generated a list of genes within 100 kb of a top 0.1% SNP and asked if this list contained more Panther pathway genes as well as HIF-1 pathway genes that would be expected by chance using a chi-square test. We corrected the pathway results for multiple testing with a Bonferroni correction. We used a range of 100 kb because we were interested in retaining potential *cis*-regulatory variants in our analysis.

#### Association testing

We performed a non-parametric Kruskal-Wallis test to test for significant differences between the high- and low-altitude population samples. We then used the software package EMMAX [30] with age and altitude as covariates to test for associations between genotypes and hemoglobin levels. The EMMAX analysis incorporates a correction for relatedness (using the identity by descent (IBS) method) within populations and structure between them via a pair-wise matrix of genetic relationships amongst individuals. Therefore, we included the entire male Amhara population sample (*n *= 28) and combined male Aari/Hamer population sample (*n *= 14). Given our modest sample size, we tested for association only with SNPs within 100 kb of a 0.1% LSBL SNP, and we used the Benjamini-Hochberg false discovery rate correction for multiple testing. Again, we used a range of 100 kb because we were interested in retaining potential *cis*-regulatory variants in our analysis. In addition, we excluded SNPs with only two genotypes present in our sample.

### Data availability

Genotyping data have been deposited in dbGaP under accession number phs000449.v1.p1

## Abbreviations

HIF: hypoxia inducible factor; iHS: integrated haplotype score; LD: linkage disequilibrium; LSBL: locus-specific branch length; SNP: single nucleotide polymorphism; XP-CLR: cross-population composite likelihood ratio.

## Competing interests

The authors declare that they have no competing interests.

## Authors' contributions

LBS performed analyses, designed the analyses and wrote the manuscript. SS, CL and JJ performed analyses. WB, AR, DWM, GB and DA assisted in study design and data collection. SAT supervised the study, assisted in study design and data collection, designed the analyses and wrote the manuscript. All authors have read and approved the manuscript for publication.

## Supplementary Material

Additional file 1 **Supplementary Tables S1 to S8**. Given their length, we are providing Supplementary Tables S1 to S8 as a separate.xls file containing eight sheets, one for each table. Click here for file

Additional file 2 **Figure S1 - hemoglobin levels associated with THRB (RS826216) genotypes**. The three THRB genotypes along the x-axis and the corresponding hemoglobin levels along the y-axis. The C/C gentoype sample size = 10, the C/T genotype sample size = 17, and the T/T genotype sample size = 15. Click here for file

Additional file 3 **Figure S2 - extended haplotype patterns at CBARA1**. Phased haplotypes at the *CBARA1 *locus for all individuals (above) and for the Omotic and Amhara individuals clustered separately (below). The chromosomal position is displayed along the x-axis and each haplotype is displayed along the y-axis. Click here for file

Additional file 4 **Figure S3 - map of the field sites**. A map of Ethiopia with each of the three field sites marked with a grey dot. Click here for file

Additional file 5 **Figure S4 - histogram of F_ST _values genome-wide**. The x-axis displays the binned F_ST _values and the y-axis displays the number of SNPs that fall in the bin. Click here for file

Additional file 6 **Figure S5 - histogram of Amhara LSBL values genome-wide**. The x-axis displays the binned LSBL values and the y-axis displays the number of SNPs that fall in the bin. Click here for file

Additional file 7 **Figure S6 - histogram of iHS values genome-wide**. The x-axis displays the binned iHS values and the y-axis displays the number of SNPs that fall in the bin. Click here for file

Additional file 8 **Figure S7 - histogram of XP-CLR values genome-wide**. The x-axis displays the binned XP-CLR values and the y-axis displays the number of SNPs that fall in the bin. Click here for file
