# Supplementary figures and images for: Genetic adaptation to high altitude in the Ethiopian highlands
Source: Genome Biol. 2012 Jan 20;13(1):R1. doi: 10.1186/gb-2012-13-1-r1 (PMC3334582; doi:10.1186/gb-2012-13-1-r1)

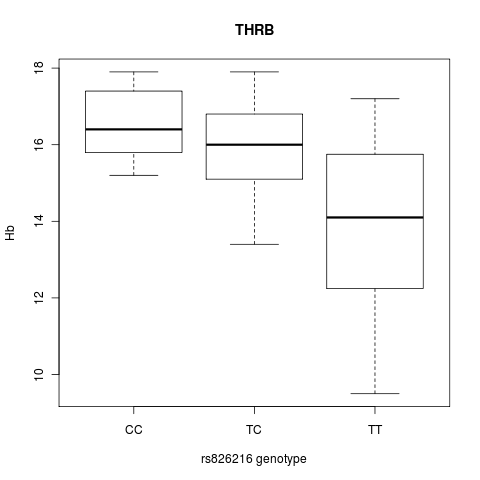

Supplement: Additional file 2 — Figure S1 - hemoglobin levels associated with THRB (RS826216) genotypes. The three THRB genotypes along the x-axis and the corresponding hemoglobin levels along the y-axis. The C/C gentoype sample size = 10, the C/T genotype sample size = 17, and the T/T genotype sample size = 15. [file gb-2012-13-1-r1-S2.PNG]

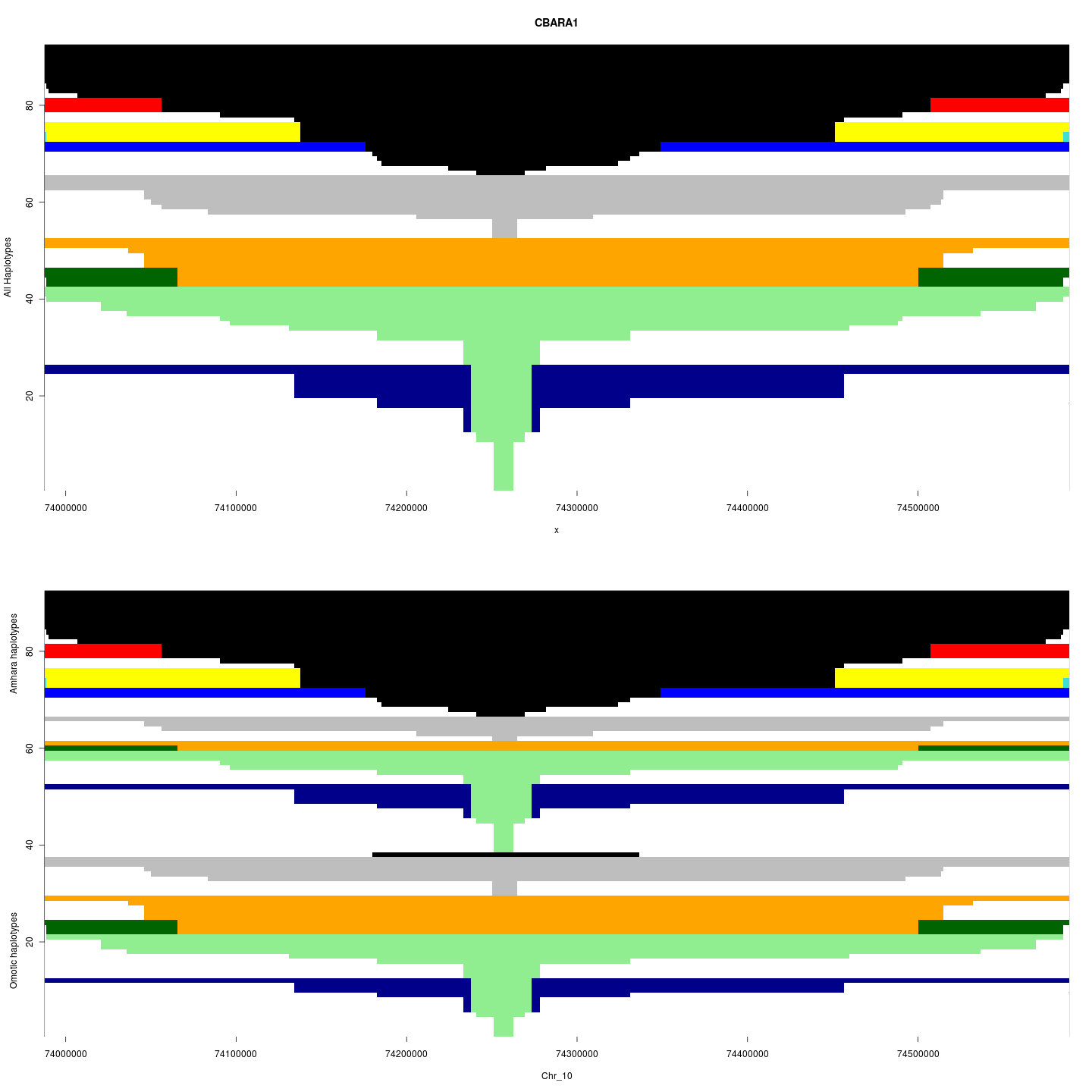

Supplement: Additional file 3 — Figure S2 - extended haplotype patterns at CBARA1. Phased haplotypes at the CBARA1 locus for all individuals (above) and for the Omotic and Amhara individuals clustered separately (below). The chromosomal position is displayed along the x-axis and each haplotype is displayed along the y-axis. [file gb-2012-13-1-r1-S3.PNG]

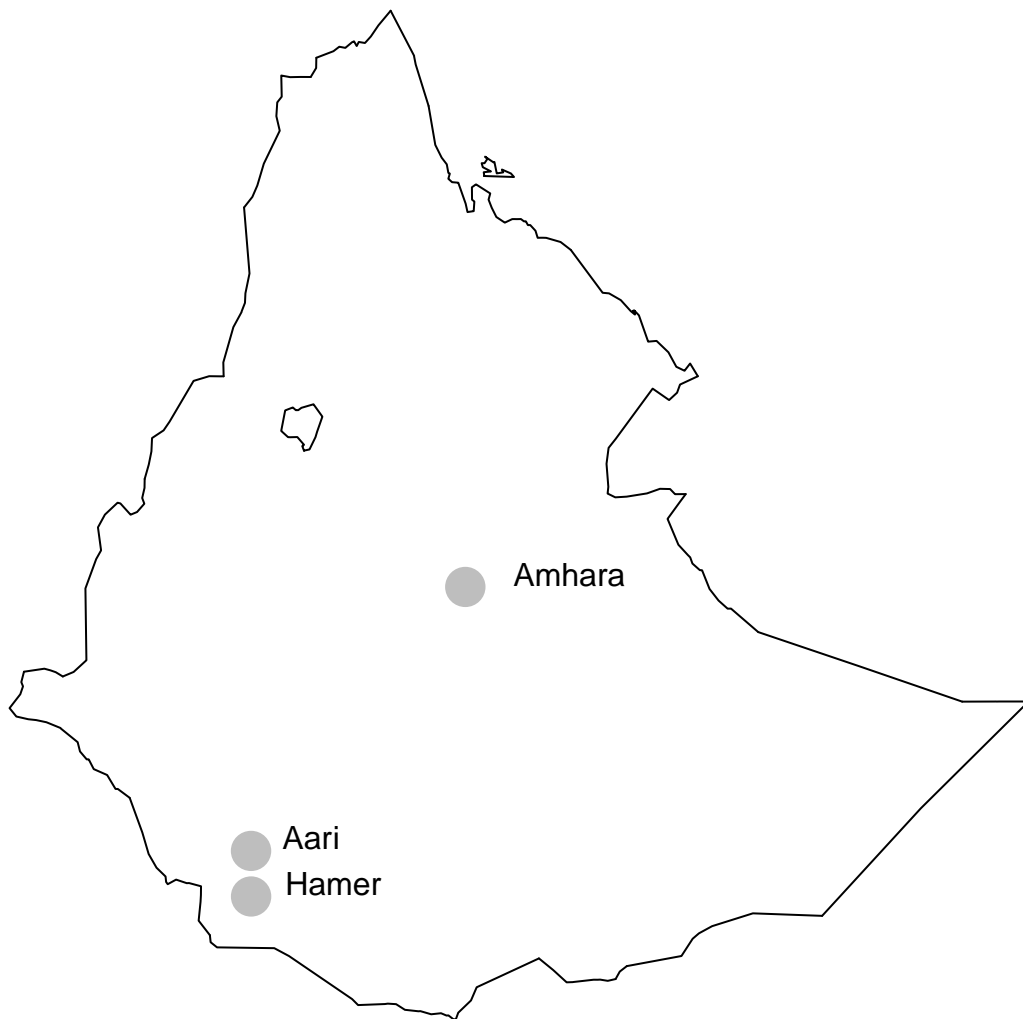

Supplement: Additional file 4 — Figure S3 - map of the field sites. A map of Ethiopia with each of the three field sites marked with a grey dot. [file gb-2012-13-1-r1-S4.PDF]

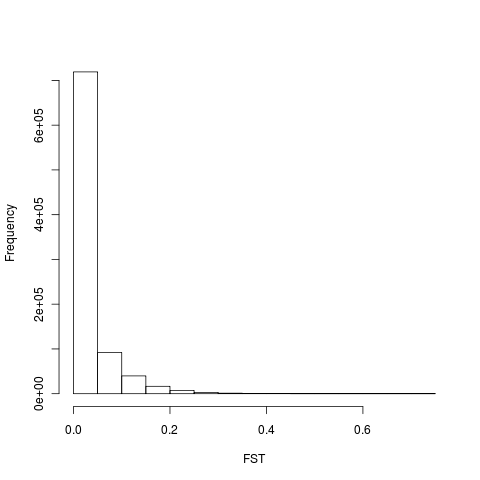

Supplement: Additional file 5 — Figure S4 - histogram of FST values genome-wide. The x-axis displays the binned FST values and the y-axis displays the number of SNPs that fall in the bin. [file gb-2012-13-1-r1-S5.PNG]

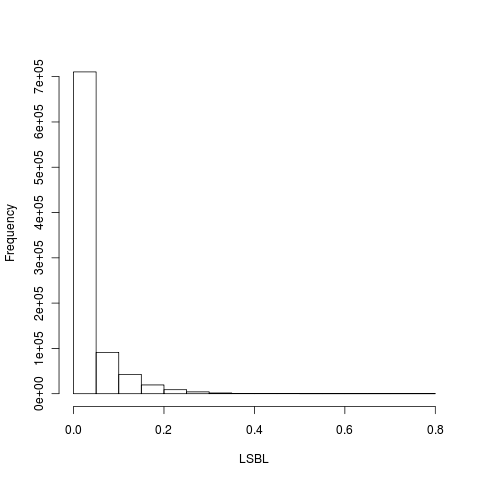

Supplement: Additional file 6 — Figure S5 - histogram of Amhara LSBL values genome-wide. The x-axis displays the binned LSBL values and the y-axis displays the number of SNPs that fall in the bin. [file gb-2012-13-1-r1-S6.PNG]

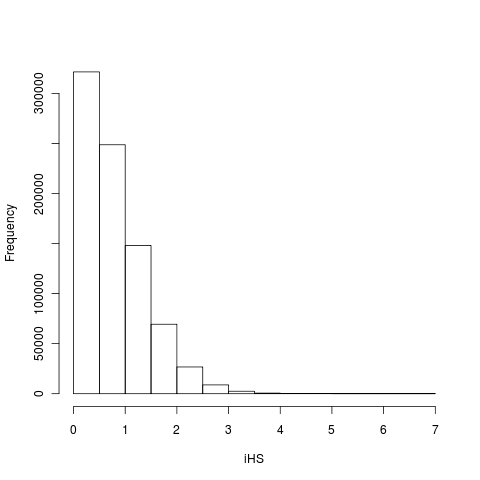

Supplement: Additional file 7 — Figure S6 - histogram of iHS values genome-wide. The x-axis displays the binned iHS values and the y-axis displays the number of SNPs that fall in the bin. [file gb-2012-13-1-r1-S7.PNG]

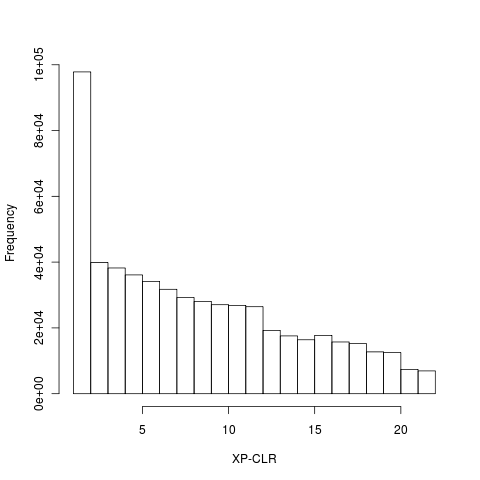

Supplement: Additional file 8 — Figure S7 - histogram of XP-CLR values genome-wide. The x-axis displays the binned XP-CLR values and the y-axis displays the number of SNPs that fall in the bin. [file gb-2012-13-1-r1-S8.PNG]
